# Maple syrup urine disease in Brazilian patients: variants and clinical phenotype heterogeneity

**DOI:** 10.1186/s13023-020-01590-7

**Published:** 2020-11-01

**Authors:** Ana Vitoria Barban Margutti, Wilson Araújo Silva, Daniel Fantozzi Garcia, Greice Andreotti de Molfetta, Adriana Aparecida Marques, Tatiana Amorim, Vânia Mesquita Gadelha Prazeres, Raquel Tavares Boy da Silva, Irene Kazue Miura, João Seda Neto, Emerson de Santana Santos, Mara Lúcia Schmitz Ferreira Santos, Charles Marques Lourenço, Tássia Tonon, Fernanda Sperb-Ludwig, Carolina Fischinger Moura de Souza, Ida Vanessa Döederlein Schwartz, José Simon Camelo

**Affiliations:** 1 Department of Pediatrics, Ribeirão Preto Medical School, University of São Paulo, Bandeirantes Av., 3900 – HC Criança - off D506, Ribeirão Prêto, SP 14049-900 Brazil; 2 Department of Genetics, Ribeirão Preto Medical School, University of São Paulo, Ribeirão Preto, SP Brazil; 3 National Institute of Science and Technology in Stem Cell, and Cell Therapy, Regional Blood Center of Ribeirão Preto, Ribeirão Preto, SP Brazil; 4 Center for Medical Genomics at Clinics Hospital of the Ribeirão Preto Medical School, University of São Paulo, Ribeirão Preto, SP Brazil; 5 Associação de Pais e Amigos dos Excepcionais of Salvador, Salvador, BA Brazil; 6 Department of Life Sciences, Bahia State University, Salvador, BA Brazil; 7 Federal University of Amazonas, Manaus, AM Brazil; 8 Department of Pediatrics, Medical Sciences School, Rio de Janeiro State University, Rio de Janeiro, RJ Brazil; 9 Sírio-Libanês Hospital, São Paulo, SP Brazil; 10 Department of Medicine, Federal University of Sergipe, São Cristóvão, SE Brazil; 11 Pequeno Príncipe Hospital, Curitiba, PR Brazil; 12 Medical School, Estácio University Center of Ribeirão Preto, Ribeirão Preto, SP Brazil; 13 Posgraduate Programme in Medicine – Medical Sciences, Federal University of Rio Grande Do Sul, Porto Alegre, RS Brazil; 14 Department of Genetics, Federal University of Rio Grande Do Sul, Porto Alegre, RS Brazil; 15 BRAIN Laboratory (Basic Research and Advanced Investigations in Neurosciences), Clinics Hospital of Porto Alegre, Porto Alegre, RS Brazil; 16 Medical Genetics Service, Clinics Hospital of Porto Alegre, Department of Genetics, Federal University of Rio Grande Do Sul, Porto Alegre, RS Brazil

**Keywords:** Inborn errors of metabolism, Maple syrup urine disease, Branched-chain amino acids, Valine, Leucine, Isoleucine

## Abstract

**Background:**

Maple syrup urine disease (MSUD) is an autosomal recessive inherited metabolic disease caused by deficient activity of the branched-chain α-keto acid dehydrogenase (BCKD) enzymatic complex. BCKD is a mitochondrial complex encoded by *BCKDHA*, *BCKDHB*, *DBT*, and *DLD* genes. MSUD is predominantly caused by Variants in *BCKDHA*, *BCKDHB*, and *DBT* genes encoding the E1α, E1β, and E2 subunits of BCKD complex, respectively. The aim of this study was to characterize the genetic basis of MSUD by identifying the point variants in *BCKDHA*, *BCKDHB*, and *DBT* genes in a cohort of Brazilian MSUD patients and to describe their phenotypic heterogeneity. It is a descriptive cross-sectional study with 21 MSUD patients involving molecular genotyping by Sanger sequencing.

**Results:**

Eight new variants predicted as pathogenic were found between 30 variants (damaging and non-damaging) identified in the 21 patients analyzed: one in the *BCKDHA* gene (p.Tyr120Ter); five in the *BCKDHB* gene (p.Gly131Val, p.Glu146Glnfs * 13, p.Phe149Cysfs * 9, p.Cys207Phe, and p.Lys211Asn); and two in the *DBT* gene (p.Glu148Ter and p.Glu417Val). Seventeen pathogenic variants were previously described and five variants showed no pathogenicity according to in silico analysis.

**Conclusion:**

Given that most of the patients received late diagnoses, the study results do not allow us to state that the molecular features of MSUD variant phenotypes are predictive of clinical severity.

## Background

Maple syrup urine disease (MSUD) (OMIM #24860), also known as leucinosis, is an inborn error of metabolism (IEM) caused by the deficiency of thiamine-dependent branched-chain α-ketoacid dehydrogenase (BCKD), composed of the subunits E1α, E1β, E2, and E3. This particular enzymatic deficiency is found in the several tissues, resulting in elevated blood plasma leves for ketoacids (α-ketoisocaproic, α-ketoisovaleric, α-keto-β-methylvaleric), alloisoleucine, and branch chain aminoacids (valine, leucine and isoleucine). Elevated levels of these amino acids mainly damage the central nervous system (CNS) [1]; in patients with MSUD, branched-chain α-ketoacids cannot be oxidized by the dehydrogenase complex and leucine tolerance reflects unmeasured protein losses and the balance between endogenous protein synthesis and degradation [2]. Some studies suggest that MSUD is characterized by an increase in oxidative stress, even in treated patients [3, 4].

Several mutations causing MSUD have already been described in the catalytic subunits of the BCKD complex encoded by four different genes. Based on altered loci, four genetic subtypes have been proposed: type Ia or E1α (MIM # 608348) for the mutations found in the *BCKDHA* (E1α subunit) gene; type Ib or E1β (MIM # 248611) for the mutations found in the *BCKDHB* (E1β subunit) gene, type II or E2 (MIM # 248610) for mutations in the *DBT* gene (E2 subunit) and type III or E3 (MIM # 238331) for mutations in the *DLD* gene (E3 subunit) [1, 5–9]. Another two different genes were more recently described how members of the BCKD complex in MSUD: *BCKDK* gene (MIM # 614923) and *PPM1K* gene (MIM # 615135); defects of PPM1K may account for a subset of human MSUD; inactivating variants of BCKDK in humans are associated with BCAA deficiency, MSUD, autism, epilepsy, and intellectual disability [10].

Although six genes (*BCKDHA*,* BCKDHB*,* BCKDK*,* DBT*,* DLD* and *PPM1K*) involved in BCAA metabolism have been reported, MSUD is predominantly caused by mutations in the *BCKDHA*, *BCKDHB*, and *DBT* genes encoding the E1α, E1β, and E2 subunits of the BCKD complex, respectively [11]. Mutations in the *DLD* gene alter the function of four different enzymes, since the E3 component is used by other mitochondrial complexes [9]. Considering the three major genes determining the disease, more than 300 mutations have been described in The Human Gene Mutation Database—HGMD to date. The HGMD currently identifies 104 mutations for the *BCKDHA* gene, 127 mutations for the *BCKDHB* gene, and 84 mutations for the *DBT* gene. In ClinVar has 211 entries only for *BCKDHA* gene and 278 for *BCKDHB* gene.

Genomic alterations that impair BCKD activity may occur in any of the three genes, but since the inheritance follows an autosomal recessive Mendelian pattern, both alleles at a single locus of one of the genes must harbor a mutation. The altered or missing protein product renders the BCKD complex inactive. Most of the mutations described affect the *BCKDHA* gene or the *BCKDHB* gene [1, 8, 9].

The standard treatment involves dietary restriction of branched-chain amino acids from natural protein and a BCAA-free amino acid specialized formula; however, liver transplantation has been performed in certain patients and may restore some enzymatic activity in MSUD patients [12], allowing diet liberalization and avoiding the occurrence of metabolic decompensation during infectious intercurrences [13]. Decreased leucine concentration has been observed hours after hepatic transplantation in patients with MSUD, using livers from cadaveric donors [12, 14–16].

The worldwide incidence of MSUD is estimated at one in 185 000 births. Although it is a rare defect, the estimated incidence of MSUD is one in 200 live births for certain Mennonite communities in Pennsylvania and various cities in the United States [1]. A study carried out in Portugal by Quental et al. [17] that analyzed cases diagnosed by mass spectrometry found an incidence of one in 86 800 live births. There is no data reported in the literature on its prevalence in the Brazilian population [17].

Since there are no common mutations, the molecular approach usually involves direct genetic sequencing. In this study is, we hypothesized that the Brazilian population has a characteristic genotypic profile which provided the basis of our objective to characterize the nucleotide variants identified as point mutations in the *BCKDHA*, *BCKDHB*, and *DBT* in a cohort of Brazilian patients diagnosed clinically and biochemically with MSUD. We also sought to describe the observed phenotypic heterogeneity in MSUD patients.

## Methods

### Patients

Peripheral blood samples were obtained from 21 patients (all patients who were added in MSUD Network from 2013 to 2015, including one pair of siblings), who belong to the MSUD Network, Brazilian Network for Assistance and Research in Maple Syrup Urine Disease, with various clinical phenotypes. The MSUD Network seeks to structure a national organization of reference for the investigation, diagnosis, management and follow-up of MSUD patients.

### Molecular analysis of genes

DNA was extracted from peripheral blood leukocytes for the molecular analysis of the three genes involved in MSUD (*BCKDHA*, *BCKDHB*, and *DBT*).

### Primer design

The forward and reverse primers of the 30 exons to be studied were designed manually using the GenBank® Reference with the following access numbers: *BCKDHA*, NM_000709.3; *BCKDHB*, NM_000056.3; and *DBT*, NM_001918.2 (https://www.ncbi.nlm.nih.gov/genbank/). All primer sequences were located in the intron regions and the amplified fragments spanned all the exons. Additional tables files shows primer sequences, PCR conditions and amplified fragment sizes in more detail (see Additional file 1 for *BCKDHA* gene, Additional file 2 for *BCKDHB* gene and Additional file 3 for *DBT* gene).

### DNA sequencing

PCR amplified fragments were sequenced on an ABI 3500xL Genetic Analyzer capillary sequencer—24-capillary DNA analysis system (Applied Biosystems, Foster City, CA, USA) using BigDye® Terminator v3.1 cycle sequencing kit (Applied Biosystems).

### Variant analysis

The sequencing results were visualized using FinchTV® version 1.4.0 software (Geospiza, Seattle, WA, USA) and compared with the relevant reference sequences from the GenBank® database (NIH, Bethesda, MD, USA). The nomenclature for the description of sequence variants detected was derived from the recommendations of the Human Genome Variation Society (https://www.hgvs.org/mutnomen) [18]. The allelic frequency of each variant was investigated in the Genome Aggregation Database (or gnomAD, data from 123,136 exomes and 15,496 genomes, people around the world) [19] and in Online Archive of Brazilian Mutations (or ABraOM, data from 609 exomes, people from the city of São Paulo, Southeast of Brazil) [20]. In order to verify the pathogenicity potential of the missense mutations, in silico analysis was performed using MutPred® v1.2 [21], Polyphen-2®-Polymorphism Phenotyping v2 software [22] and SIFT® [23]. Sequence variants were evaluated for their disease-causing potential using the Mutation Taster application [24].

### Clinical data analysis

Clinical data analysis results of information such as age at diagnosis, family history of the disease, consanguinity, results of the first dosage levels of the branched-chain amino acids (at diagnosis), neurodevelopment delay, and phenotypic classification of the patients were obtained from the registry of clinical records standardized by the Brazilian MSUD Network, which was conducted by the professional responsible for patient care at the Reference Center.

## Results

The *BCKDHA*, *BCKDHB*, and *DBT* genes from 21 patients (52.4% female), including one pair of siblings, were studied. Eight new variants predicted as pathogenic were found between 30 variants (damaging and non-damaging) identified in the 21 patients analyzed: one in the *BCKDHA* gene, five in the *BCKDHB* gene and two in the *DBT* gene. Another seventeen pathogenic variants found in Brazilian patients have been reported in the literature: five in the *BCKDHA* gene, eleven in the *BCKDHB* gene, and one in the *DBT* gene. It can be seen that most of the mutations found in the *BCKDHA* gene were in exons 2, 3, and 4 and in *BCKDHB* gene, mainly in exons 4 and 5, characterizing hot spots.

Among the 21 patients, six were homozygotes for a pathogenic allele. In 18 of the 21 patients, only one pathogenic allele could be identified. We also identified five non-pathogenic variants among our cohort (Tables 1, 2).

**Table 1:** Clinical, biochemical and molecular data of Brazilian MSUD patients

ID	Gender	Age at diagnosis	Plasma isoleucine (μMol/L) at diagnosis	Plasma leucine (μMol/L) at diagnosis	Plasma valine (μMol/L) at diagnosis	Time from diagnosis to onset of dietary manegement	Outcome	Family history	Consanguinity	Clinical phenotype	Genetic subtype	Gene	Type	Mutation at nucleotide level cDNA
1^a,b^	Female	60d	699.5	3252.5	999.5	60d	Severe DD + died	No	Yes	Classic	EIb	*BCKDHB*	Dup	c.[92_102dup11]; [92_102dup11]
2^c,d^	Female	30d	45.7	278.9	35.1	NA	Severe DD + died	Yes	Yes	Classic	EII	*DBT*	M	c.[346G > A];[346G > A]
3^c^	Male	15d	224.4	2646.1	371.1	15d	Slight DD	Yes	Yes	Classic	EII	*DBT*	M	c.[346G > A];[346G > A]
4	Male	26d	149.0	2037.8	263.3	1d	Moderate DD	No	No	Classic	EIb	*BCKDHB*	D	c.[595_596delAG];[595_596delAG]
5^e,f^^,j^	Male	17d	207.0	2914.0	304.5	20d	Slight DD	NA	NA	Classic	EIb	*BCKDHB*	D/I + D + M + M	c.[**436delinsCA**; **445_446delTT**];[**620G > T**; **633G > C**]
6^f^	Female	17d	482.7	1484.0	942.1	3d	Normal neurodevelopment	No	No	Classic	EIb	*BCKDHB*	M + D	c.[498G > C];[595_596delAG]
7	Female	16m	333.0	1503.1	938.9	NA	Slight DD	No	No	Intermediate	EII	*DBT*	N	c.[**442G > T**];[**442G > T**]
8^j^	Female	26d	179.5	1488.7	235.1	15d	Severe DD	Yes	No	Classic	EIb	*BCKDHB*	M + D + D	c.[506A > G]; [743delC; 764_840 + 4del; 840 + 9_ + 22del]
9	Female	60d	1336.0	5002.0	979.0	15d	Severe DD	No	No	Classic	EIb	*BCKDHB*	N + ?	c.[853C > T];[?]
10^g,h^	Female	NA	NA	NA	NA	NA	NA	NA	NA	NA	EIa	*BCKDHA*	N + SS	c.[859C > T];[109-3575_288 + 4del]
11^j^	Male	17d	340.1	2000.0	409.1	2d	Slight DD	No	Yes	Classic	EIb	*BCKDHB*	D	c.[743delC**;** 764_840 + 4del; 840 + 9_ + 22del]**;** [743delC**;** 764_840 + 4del; 840 + 9_ + 22del]
12^g^	Male	30d	25.4 (Ile + Leu)		3.7	60d	Severe DD	No	NA	Classic	Eia	*BCKDHA*	I + ?	c.[740_741insT];[?]
13^g^	Male	30d	31.0	904.1	406.6	20d	Moderate DD	Yes	No	Classic	EIb	*BCKDHB*	M + D	c.[502C > T];[595_596delAG]
14^a^	Male	NA	NA	NA	NA	NA	NA	NA	NA	NA	EIb	*BCKDHB*	M + M	c.[605C > T];[641 T > A]
15^i^	Male	24 m	895.4	1782.2	1038.2	NA	NA	No	NA	NA	Eia	*BCKDHA*	M	c.[659C > T**]**;[659C > T]
16^a^	Female	NA	NA	NA	NA	NA	NA	NA	NA	NA	EIb	*BCKDHB*	M	c.[**392G > T**];[**392G > T**]
17^g^	Female	35d	1700.0	1700.0	NA	10d	Severe DD	No	NA	Classic	EIb	*BCKDHB*	D + SS	c.[595_596delAG];[343 + 2 T > G]
18	Male	3d	755.0 (Ile + Leu)		345.0	0d	NA	Yes	No	NA	EIb	*BCKDHB*	M + M	c.[**392G > T**];[641 T > A]
19^a^	Female	NA	NA	NA	NA	NA	NA	NA	NA	NA	EII	*DBT*	N	c.[**442G > T**];[**442G > T**]
20	Female	14y	220.8	564.6	493.3	NA	Moderate or severe DD	No	No	Intermediate	EIa	*BCKDHA*	D + M	c.[**357_358delCT**];[454G > A]
21	Male	12y	151.2	580.9	444.3	2y	Moderate DD	No	No	Intermediate or thiamine responsive	EIa	*BCKDHA*	I + ?	c.[740_741insT];[?]

Novel Mutations are highlighted in bold

*ID* patient identification, *m* months, *d* days, *a* years, *DD* developmental disability, *NA* data not available, *M* missense, *Dup* duplication, *D* deletion, *I* insertion, *N* nonsense, *SS* splice site

^a^Patient also carries the non disease-causing variation c. 116C > A in one *BCKDHA* allele (p.Pro39His) and the non disease-causing variation c.166G > C in one *BCKDHA* allele (p.Gly56Arg)

^b^Patient also carries the non disease-causing variation c.188_189delinsCA in one *BCKDHB* allele (p.Arg63Pro) and the damaging variation c.92_102dup11 in two *BCKDHB* alleles (p.Phe35Trpfs * 41)

^c^Siblings

^d^Patient also carries the non disease-causing novel variation c.1250A > T in one *DBT* allele (p.Glu417Val), probably a polymorphism in this case

^e^Patient also carries the non disease-causing variation c.116C > T in one *BCKDHA* allele (p.Pro39Leu)

^f^Patient also carries the non disease-causing variation c.1150A > G in one *DBT* allele (p.Ser384Gly)

^g^Liver Transplant

^h^Patient also carries the non disease-causing variation c. 116C > A in two *BCKDHA* alleles (p.Pro39His)

^i^Patient also carries the non disease-causing variation c.166G > C in one *BCKDHA* allele (p.Gly56Arg)

^j^Patient carries more than 2 variants, but without parental testing, it is impossible to determine which of the variants are in cis or trans configuration and what the genotype is (disease-causing and non disease-causing variations, exactly)

**Table 2:** Variations detected in type Ia, type Ib and type II Brazilian MSUD patients

Spot	Nucleotide change	Protein prediction	Type	Prediction of pathogenicity			Classification	ACMG classification	Reference^b^	Frequency (patients)
				SIFT®	Polyphen-2®	MutPred®				
BCKDHA gene										
Promoter	c.109-3575_288 + 4del	p.(?)	SS	–	–	–	Damaging	–	Feier et al. [15]	1/21
Exon 2	c.116C > A	p.(Pro39His)	M	Non-damaging	Non-damaging	Non-damaging	Non-damaging	–	Henneke et al. [25]^c^	5/21
Exon 2	c.116C > T	p.(Pro39Leu)	M	Non-damaging	Non-damaging	Non-damaging	Non-damaging	Uncertain significance PP2/PP3/PM2	This study^a^	1/21
Exon 2	c.166G > C	p.(Gly56Arg)	M	Damaging	Damaging	Non-damaging	Non-Damaging	Uncertain significance PP2/PP3/PM2	This study	2/21
Exon 3	c.357_358delCT	p.(Tyr120Ter)	D	–	–	–	Damaging	Phatogenic PVS1/PP3/PM2	This study	1/21
Exon 4	c.454G > A	p.(Asp152Ans)	M	Damaging	Damaging	Damaging	Damaging	–	Quental et al. [17]	1/21
Exon 6	c.659C > T	p.(Ala220Val)	M	Damaging	Damaging	Damaging	Damaging	–	Rodriguez-Pombo et al. [9]	1/21
Exon 6	c.740_741insT	p.(Ala248Cysfs * 10)	I	–	–	–	Damaging	–	DB	2/21
Exon 7	c.859C > T	p.(Arg287Ter)	N	–	–	–	Damaging	–	Chinsky et al. [37]	1/21
BCKDHB gene										
Exon 1	c.92_102dup11	p.(Phe35Trpfs * 41)	Dup	–	–	–	Damaging	–	Rodriguez-Pombo et al. [9]	1/21
Exon 1	c.188_189delinsCA	p.(Arg63Pro)	M	Non-damaging	Non-damaging	Non-damaging	Non-damaging	Uncertain significance BP4/PM2/PP2	This study	1/21
Intron 3	c.343 + 2 T > G	p.(?)	SS	–	–	–	Damaging	–	Feier et al. [15]	1/21
Exon 4	c.392G > T	p.(Gly131Val)	M	Damaging	Damaging	Damaging	Damaging	Uncertain significance PP2/PP3/PM2	This study	2/21
Exon 4	c.436delinsCA	p.(Glu146Glnfs * 13)	D/I	–	–	–	Damaging	Pathogenic PVS1/PP3/PM2	This study	1/21
Exon 4	c.445_446delTT	p.(Phe149Cysfs * 9)	D	–	–	–	Damaging	Pathogenic PVS1/PP3/PM2	This study	1/21
Exon 5	c.498G > C	p.(Lys166Asn)	M	Damaging	Damaging	Damaging	Damaging	–	Scaini et al. [38]	1/21
Exon 5	c.502C > T	p.(Arg168Cys)	M	Damaging	Damaging	Damaging	Damaging	–	Flaschker et al. [34]	1/21
Exon 5	c.506A > G	p.(Tyr169Cys)	M	Damaging	Damaging	Damaging	Damaging	–	DB	1/21
Exon 5	c.595_596delAG	p.(Pro200Ter)	D	–	–	–	Damaging	–	Henneke et al. [25]	4/21
Exon 5	c.605C > T	p.(Ala202Val)	M	Damaging	Damaging	Damaging	Damaging	–	DB	1/21
Exon 5	c.620G > T	p.(Cys207Phe)	M	Damaging	Damaging	Non-damaging	Damaging	Uncertain significance PP2/PP3/PM2	This study^a^	1/21
Exon 5	c.633G > C	p.(Lys211Asn)	M	Damaging	Damaging	Damaging	Damaging	Uncertain significance PP2/PP3/BP6	This study^a^	1/21
Exon 6	c.641T > A	p.(Ile214Lys)^a^	M	Damaging	Damaging	Damaging	Damaging	–	Rodriguez-Pombo et al. [9]	2/21
Exon 7	c.743delC	p.(Ala248Glyfs * 6)	D	–	–	–	Damaging	–	Feier et al. [15]	2/21
Exon 7 + Intron 7	c.764_840 + 4del;840 + 9_ + 22del^f^	?	D	–	–	–	Damaging	–	Feier et al. [15]	2/21
Exon 8	c.853C > T	p.(Arg285Ter)^e^	N	–	–	–	Damaging	–	Henneke et al. [25]	1/21
DBT gene										
Exon 4	c.346G > A	p.(Gly116Arg)	M	Damaging	Damaging	Damaging	Damaging	–	Scaini et al. [38]	2/21
Exon 5	c.442G > T	p.(Glu148Ter)	N	–	–	–	Damaging	Pathogenic PVS1/PP3/PP5/PM2	This study	2/21
Exon 9	c.1150A > G	p.(Ser384Gly)	M	Non-damaging	Non-damaging	Non-damaging	Non-damaging	–	Tsuruta et al. [35]^d^	2/21
Exon 10	c.1250A > T	p.(Glu417Val)	M	Damaging	Damaging	Damaging	Damaging	Uncertain significance PP3/PM2	This study	1/21

*M* missense, *Dup* duplication, *D* deletion, *I* insertion, *N* nonsense, *SS* splice site, *DB* database

^a^New mutation related to MSUD, however presented in data banks

^b^Mutation Database HGMD (*The Human Gene Mutation Database*) and *Mutation Taster*

^c^The mutation is probably a polymorphism, previously named T106M (Dursun et al. [39])

^d^Non damaging mutation previously named G323S (Tsuruta et al. [35])

^e^The variation of nucleotide sequency have not been identified at the genomic level; at cDNA level, the effect is the 3’UTR site alteration

^f^We have also identified these two variants occurring together

Among pathogenic variants in our patient cohort, the Pro200Ter in the *BCKDHB* gene was the most prevalent (19.0% of the patients, n = 4, one of whom was homozygous). Among non-pathogenic variants (per in silico analysis), the Pro39His variant in the *BCKDHA* gene was the most frequent (23.8% of the patients, n = 5) and was always found in the setting of two other pathogenic variants that explains the MSUD phenotype.

Of the 24 pathogenic variants found in the cohort of patients studied, the most prevalent type of pathogenic mutation was missense (n = 13). There were eight variants that exhibited changes in the codon-scoring matrix or frameshift, with five resulting from deletion (four in the *BCKDHB* gene and one in the *BCKDHA* gene), one resulting from insertion into the *BCKDHA* gene, one resulting from deletion and insertion into the *BCKDHB* gene, and one resulting from a duplication in the *BCKDHB* gene. The least frequent type was nonsense (n = 3, one variant in each gene studied); the next two least frequent types (frameshift and nonsense mutations) truncate the protein prematurely by the presence of the terminator codon and are considered pathogenic a priori.

Regarding possible genotype–phenotype associations, out of 12 patients exhibiting the classical clinical phenotype, nine presented the genetic subtype EIb, two presented the genetic subtype EII and one presented the genetic subtype EIa. The two patients with intermediate clinical phenotype had different genetic subtypes (EIa and EII) and the EIa genetic subtype was detected in the patient with intermediate or thiamine responsive phenotype. Hence, there is likely no consistent association between clinical phenotypes with the genetic subtypes. However, it is thought that mutations at different locations in the three genes can trigger clinical signs and varied sequelae; these mutations are also classically described in the literature for other diseases characterized by great variability in frequency and severity of clinical manifestations and complications, suggesting, for example, a worse prognosis in the presence of a localized mutation in the gene’s functional domain (Table 1).

Evaluating the genetic subtype of the patients who presented EIa, the phenotype was extremely varied (one patient had a classical phenotype, one intermediate, and one intermediate or thiamine responsive); eight EIb patients presented classical phenotype; EII cases were classical (n = 2) and intermediate (n = 1) phenotypes (Table 1).

## Discussion

To date, this is the first study in Brazil to identify variants in the *BCKDHA*, *BCKDHB*, and *DBT* genes in clinically and biochemically diagnosed MSUD patients enrolled in the MSUD Network. We found that out of the 42 alleles, the disease-causing variant was located in the *BCKDHA* gene in 23.8% (5 patients), in the *BCKDHB* gene in 57.1% (12 patients), and in the *DBT* gene in 19.1% (4 patients).

Some of the novel variants have already been detected in database projects involving the search for variants in a large number of individuals but never related to patients. We investigate the variants in “The Exome Aggregation Consortium”—ExAC—composed of 60,706 unrelated individuals, and the Online Archive of Brazilian Mutations—AbraOM—composed of 609 elderly individuals [19, 20]. Three of 11 novel variants were in ExAC, all in very low frequencies (Table 2).

Nellis and Danner [7] studied 63 patients diagnosed clinically with MSUD and found the following frequency of mutations: 33% in the *BCKDHA* gene, 38% in the *BCKDHB* gene, and 19% in the *DBT* gene. In agreement with the results of the present study, most patients (of varied ethnicity) evaluated by Henneke et al. [25] presented mutations of the subtype Ib, followed by Ia, with the least frequent mutation occurring in the *DBT* gene (subtype II). In a cohort of 32 unrelated Turkish patients, out of 64 alleles, the disease-causing mutation was located in the *BCKDHA* gene in 37% (12 patients), in the *BCKDHB* gene in 44% (14 patients), and in the *DBT* gene in 19% (6 patients) [26]. However, Quental et al. [6], in a study with 30 Portuguese patients, found 17 mutations with no difference in the frequency of these genes, with six in the *BCKDHA* gene, five in the *BCKDHB* gene, and six in the *DBT* gene. A study published by Abiri et al. [27] investigating 20 Iranian families of MSUD patients, six patients demonstrated homozygous haplotype for the *BCKDHA* gene, nine for the *BCKDHB* gene, and two for the *DBT* gene. Gupta et al. [28], studying 24 unrelated Indian patients, found 20 mutations in 22 of the patients, with 11 novel mutations—four in the *BCKDHA* gene, six in the *BCKDHB* gene, with a novel mutation identified in the *DBT* gene.

Li et al. [29] described eight cases of MSUD (in four females and four males) from unrelated Chinese families who were diagnosed through serum BCAA and genetic analysis between 9 days to 1 year and 8 months of life (six patients presented the neonatal form of the disease); 12 different mutations were found with six in the *BCKDHA* gene, five in the *BCKDHB* gene, and one in the *DBT* gene, of which only one mutation located in the *BCKDHA* gene had been reported in the literature.

In agreement with the present study, all the Spanish patients studied (belonging to a cohort of 33 MSUD patients) by Rodríguez-Pombo et al. [9] who had a mutation in the *BCKDHB* gene (subtype Ib) also had the classical phenotype; however, inconsistencies were found between the biochemical parameters of the patients and their clinical phenotype, as in the case of three patients who were homozygotic for the same mutation and exhibited a residual enzymatic activity variable between < 1 and 13%.

Abiri et al. [30] reported six Iranian patients who were homozygous for mutations in the *BCKDHA* gene, demonstrating that most of the mutations found were in exons 2, 4, and 6. Recently, 20 new mutations were identified in the *BCKDHA*, *BCKDHB*, and *DBT* genes of 52 MSUD patients from Saudi Arabia, with no mutation in the *DLD* gene identified [31].

The leucine levels at diagnosis were high and are, in the majority of the studied patients, considered critical, and can produce irreversible damage or even death of the patient [32]. Leucine can increase rapidly during catabolic states, altering brain chemistry by competing with nine other amino acids for entry into the brain via the facilitative SLC7A5 transporter [2]. Branched-chain amino acid transaminase (BCAT1) catalyzes the formation of α-ketoisocaproic acid (αKIC) from leucine and α-ketoglutarate (αKG); αKIC enters brain (via the monocarboxylate transporter) and is neurotoxic at high concentrations [2]. Elevated tissue αKIC reverses normal flow through BCAT1, depletes tissues of glutamate (a substrate for glutamine and γ-aminobutyric acid—GABA), and indirectly drives flux through glutamate-pyruvate transaminase to form pyruvate from αKG and alanine [2].

According to Strauss et al. [2], in classic MSUD patients, these interconversions explain inverse relationships of leucine to glutamate, glutamine and alanine, and likely underlie the depletion of glutamate and elevation of lactate observed in brain tissue during metabolic encephalopathy. However, the results of a Brazilian study [33] on the MSUD panorama over the last 2 decades in the country did not indicate a significant association between the severity of developmental delay and leucine levels at diagnosis; this can be attributed to the fact that early diagnosis and long-term metabolic control are considered more decisive factors for psychomotor and cognitive development than leucine levels at the time of diagnosis. Therefore, regarding the characteristics and clinical situation of the patients, it is thought that the association of phenotype and genotype is not ideal in that neurological deterioration is directly associated with delayed diagnosis and thus the absence of appropriate nutritional support, which is essential for the control of BCAA serum levels as well as those of their metabolites (metabolic control).

Although some patients were diagnosed in the first month of life, as can be seen in Table 1, diagnosis was not made at the beginning of the presentation of symptoms in the majority of patients (only patients 6, 11, and 15 were diagnosed at the time at which symptoms appeared and patient 18 was diagnosed when still asymptomatic). In Brazil, the time between diagnosis and receipt of the metabolic formula is long and variable [33], which can also be observed in the registry of the clinical files of patients in our study. Only four patients (patients 4, 6, 11, and 18) received the metabolic formula for treatment of MSUD immediately after diagnosis; receipt varied for the other patients between 10 days and 2 months after diagnosis, delaying appropriate treatment of the disease. When patients are diagnosed in the acute phase of the disease during a hospital stay due to metabolic decompensation, treatment with a metabolic formula is started at the time of diagnosis only if the formula is available at the admitting hospital. In contrast, patients diagnosed in the non-acute phase of the disease and treated on an outpatient basis are prone to receive the formula late, with the guarantee of access to the metabolic formula occurring only through judicial measures [33]. These facts reinforce the need to include neonatal screening for MSUD in the Brazilian Newborn Screening Program.

Molecular genetics analysis is critical for the accurate diagnosis of MSUD patients, can aid in identifying variant phenotypes and offer guidance on prognosis and treatment. In addition, the molecular screening of affected patients provides epidemiological data for implementation of the test in populations, facilitating prenatal diagnosis in families at risk [28, 30].

Flaschker et al. [34], who described mutations in 15 individuals from Germany, Austria, and Switzerland with different phenotypic variants of MSUD, concluded that in the cohort of patients analyzed, the most severe enzymatic and clinical phenotypes of the disease forms (MSUD variants) were associated mainly with specific genotypes in the *BCKDHA* gene, whereas milder enzymatic and clinical phenotypes were associated with specific genotypes in the *BCKDHB* and *DBT* genes; the results of this study led to the conclusion that genotyping may be predictive of metabolic and clinical phenotype and may have a prognostic value, particularly in individuals with a variant of MSUD identified in neonatal screening such that early treatment will effectively delay the natural course of the disease.

However, in a study by Gupta et al. [28], it was not possible to establish any genotype and phenotype correlation in Indian MSUD patients; most of the cases (66.6%) had the classic neonatal type of MSUD, most of the classic neonatal patients (10 of 14 cases) having mutations in the *BCKDHB* gene.

Patients with the intermittent form of the disease, evaluated by Tsuruta et al. [35], presented mutations exclusively in the *DBT* gene, giving rise to low but significant residual activity of the BCKD complex. However, in the present study, no patient with the genetic subtype EII presented this phenotypic form of the disease.

Wang et al. [36] suggested that DNA sequencing associated with in silico analysis is a simple and rapid method to predict the severity of MSUD and that neonatal screening has been demonstrated to be an effective approach for screening MSUD patients in a diverse ethnic population [34], allowing early intervention with dietary therapy [36]. The identification of different missense-type mutations provides valuable evidence for the accuracy of the analysis, as well as the determination of its effect on the catalytic subunit. DNA sequencing analysis, together with in silico analysis, has been suggested to predict the clinical manifestations of MSUD, since it is a simple and rapid method [36].

According to Feier et al. [14], liver transplantation provides metabolic control, allowing the patient to resume a normal diet and avoid further neurological damage, helping the individual's neurodevelopment as well as eliminating the chance of cerebral edema and death. In this case report, Feier et al. [14] described the situation of a 2-year-old child with MSUD who underwent liver transplantation (domino transplantation); the donor was the patient’s mother. The use of living relative donors has been controversial because parents are mandatory heterozygotes, but in this case, the data suggest that the use of a donor associated with MSUD was effective. Although the traditional treatment during the maintenance phase is basically the dietary restriction of BCAA and supplementation with thiamine and a food formula without BCAA, liver transplantation is indicated in the literature as a good treatment option [14, 15, 27].

No somatic cell complementation studies were performed to assign enzymatic subtypes Ia, Ib or II, which can be a limitation. Another limitation of this study is about the ability or lack thereof of performing deletion/duplication analysis at the exon or whole gene level, and this can be related to not being able to make a molecular diagnosis in some of the patients studied. In addition, other molecular characteristics (type and location of mutation) were not considered in this analysis of this study.

## Conclusions

The point variants identified in the *BCKDHA*, *BCKDHB*, and *DBT* genes of the cohort of Brazilian patients diagnosed clinically and biochemically with the MSUD did not present a similar pattern or frequency among the genes. Eight new variants predicted as pathogenic were identified: one in the *BCKDHA* gene, five in the *BCKDHB* gene and two in the *DBT* gene. We detected a broad spectrum of clinical presentation in the cohort of Brazilian patients with MSUD, and there was no apparent association between phenotypic characteristics and the genetic subtype. Given that most Brazilian patients were diagnosed late and did not receive immediate treatment, the study results indicate no consistent relationship between molecular and clinical MSUD phenotype; however, we speculate that genetic screening may have prognostic value particularly in individuals with MSUD variants identified in expanded newborn screening, in which early treatment can likely alter the natural course of the disease.

## Supplementary information

**Additional file 1.** Primers sequences, PCR conditions and amplified fragment sizes of the BCKDHA gene. Description of data: Primer location, primer sequences, Annealing Temperature and amplified fragment sizes (amplicon size) of the BCKDHA gene.

**Additional file 2.** Primers sequences, PCR conditions and amplified fragment sizes of the BCKDHB gene. Description of data: Primer location, primer sequences, Annealing Temperature and amplified fragment sizes (amplicon size) of the BCKDHB gene.

**Additional file 3.** Primers sequences, PCR conditions and amplified fragment sizes of the DBT gene. Description of data: Primer location, primer sequences, Annealing Temperature and amplified fragment sizes (amplicon size) of the DBT gene.

## Data Availability

All data generated or analysed during this study are included in this published article. For more information, the datasets used and/or analysed during the current study are available from the corresponding author on reasonable request.

## Abbreviations

BCAA: Branched-chain amino acids
BCAT1: Branched-chain amino acid transaminase
BCKD: Branched-chain α-keto acid dehydrogenase
BCKDHA: Branched chain keto acid dehydrogenase E1, alpha polypeptide
BCKDHB: Branched chain keto acid dehydrogenase E1, beta polypeptide
CNS: Central nervous system
DBT: Dihydrolipoamide branched chain transacylase E2
DLD: Dihydrolipoamide dehydrogenase
DNA: Desoxirribonucleic acid
IEM: Inborn error of metabolism
MSUD: Maple syrup urine disease
PCR: Polimerase chain reaction
αKG: α-Ketoglutarate
αKIC: α-Ketoisocaproic acid
