# Supplementary material for: Maple syrup urine disease in Brazilian patients: variants and clinical phenotype heterogeneity
Source: Orphanet J Rare Dis. 2020 Nov 1;15:309. doi: 10.1186/s13023-020-01590-7 (PMC7603684; doi:10.1186/s13023-020-01590-7)
Supplement: Supplementary file 1 — Additional file 1. Primers sequences, PCR conditions and amplified fragment sizes of the BCKDHA gene. Description of data: Primer location, primer sequences, Annealing Temperature and amplified fragment sizes (amplicon size) of the BCKDHA gene. [file 13023_2020_1590_MOESM1_ESM.docx]

Primers sequences, PCR conditions and amplified fragment sizes of the *BCKDHA* gene.

| **Primer location** | **Primer Name** | **Primer sequence** | **AT** | **Amplicon size (bp)** |
| --- | --- | --- | --- | --- |
| Exon 1 | BCKDHA_EX1_F | 5’-CTGGTCAGGTTGCCCTCTT-3’ | 60ºC | 421 |
|  | BCKDHA_EX1_R | 5’-GGACCCCACACTCTGAAGATAG-3’ |  |  |
| Exons 2-3 | BCKDHA_EX2_3F | 5’-CACATGCTCAACCACCATG-3’ | 62ºC | 561 |
|  | BCKDHA_EX2_3R | 5’-CTTGGGAGCCATTCCTTTG-3’ |  |  |
| Exon 4 | BCKDHA_EX4_F | 5’-ACAGCAACTCGATCCCTCTG-3’ | 60ºC | 394 |
|  | BCKDHA_EX4_R | 5’-CTGCTCCTGGAAGAACACTCA-3’ |  |  |
| Exon 5 | BCKDHA_EX5_F | 5’-CTTTCCTGTCTGCCTGCC-3’ | 62ºC | 392 |
|  | BCKDHA_EX5_R | 5’-AGCACAGACCAGGGCTCTAG-3’ |  |  |
| Exon 6 | BCKDHA_EX6_F | 5’-CATCAGGAGCTGAGGTGTTTC-3’ | 60ºC | 460 |
|  | BCKDHA_EX6_R | 5’-ACAGGACGAGAACCAGGAAG-3’ |  |  |
| Exon 7 | BCKDHA_EX7_F | 5’-GGAGTTGAGGTCCTGAGCAC-3’ | 60ºC | 406 |
|  | BCKDHA_EX7_R | 5’-AGGAGGAGTGGAAACGGAAT-3’ |  |  |
| Exon 8 | BCKDHA_EX8_F | 5’-ACTGACAGCCACCGTAGCAT-3’ | 62ºC | 441 |
|  | BCKDHA_EX8_R | 5’-GGTGTTCCACAAATCCTTCC-3’ |  |  |
| Exon 9 | BCKDHA_EX9_F | 5’-GAGTGGTTAATTCCTTGCCAAG-3’ | 60ºC | 684 |
|  | BCKDHA_EX9_R | 5’-ACTCCAGGAAACAAAGACCAG-3’ |  |  |

AT: Annealing Temperature*;* F: Forward; R: Reverse*.*
