# Supplementary material for: Maple syrup urine disease in Brazilian patients: variants and clinical phenotype heterogeneity
Source: Orphanet J Rare Dis. 2020 Nov 1;15:309. doi: 10.1186/s13023-020-01590-7 (PMC7603684; doi:10.1186/s13023-020-01590-7)
Supplement: Supplementary file 2 — Additional file 2. Primers sequences, PCR conditions and amplified fragment sizes of the BCKDHB gene. Description of data: Primer location, primer sequences, Annealing Temperature and amplified fragment sizes (amplicon size) of the BCKDHB gene. [file 13023_2020_1590_MOESM2_ESM.docx]

Primers sequences, PCR conditions and amplified fragment sizes of the *BCKDHB* gene.

| **Primer location** | **Primer Name** | **Primer sequence** | **AT** | **Amplicon size (bp)** |
| --- | --- | --- | --- | --- |
| Exon 1 | BCKDHB_EX1_F | 5’-CTAGCCCACACTTCCCCTCT-3’ | 63ºC | 552 |
|  | BCKDHB_EX1_R | 5’-GCAAGGAGGTTCCAGAGAGTC-3’ |  |  |
| Exon 2 | BCKDHB_EX2_F | 5’-CTCCAGGTCTGTATTGCTTTTGT-3’ | 60ºC | 383 |
|  | BCKDHB_EX2_R | 5’-GCCCCAATCATACCTTTGAA-3’ |  |  |
| Exon 3 | BCKDHB_EX3_F | 5’-GTGTGTGTGGTAACTGTCATCCA-3’ | 60ºC | 684 |
|  | BCKDHB_EX3_R | 5’-CCCAACAGGCAGAATCTCC-3’ |  |  |
| Exon 4 | BCKDHB_EX4_F | 5’-CCTGTTCTATACTTCTCCATCCCA-3’ | 60ºC | 307 |
|  | BCKDHB_EX4_R | 5’-GGGTAGCGGCAATACTTGAA-3’ |  |  |
| Exon 5 | BCKDHB_EX5_F | 5’-AGGAAGAACGGAAGGAGATTG-3’ | 60ºC | 412 |
|  | BCKDHB_EX5_R | 5’-AACTGGGCATTGGATAGCATA-3’ |  |  |
| Exon 6 | BCKDHB_EX6_F | 5’-GCCCTTCTTAGCAGCGAGT-3’ | 60ºC | 405 |
|  | BCKDHB_EX6_R | 5’-CAGATTTCCTCTTTGTTTCCACA-3’ |  |  |
| Exon 7 | BCKDHB_EX7_F | 5’-GCACAAGTGTCACCTCAGAAAA-3’ | 60ºC | 482 |
|  | BCKDHB_EX7_R | 5’-ATAGATCTGAAGTGTCCTCGCC-3’ |  |  |
| Exon 8 | BCKDHB_EX8_F | 5’-CTCCATGCAGATCAGTTCCTG-3’ | 60ºC | 484 |
|  | BCKDHB_EX8_R | 5’-GCATAAAGGACCCCATTTTGTA-3’ |  |  |
| Exon 9 | BCKDHB_EX9_F | 5’-CCTGTCGAAAGCGAGTTGTAAC-3’ | 60ºC | 311 |
|  | BCKDHB_EX9_R | 5’-CTTCTGGAATTGGCATGTGG-3’ |  |  |
| Exon 10 | BCKDHB_EX10_F | 5’-CGAACATGCTGTTACCTGCTT-3’ | 60ºC | 391 |
|  | BCKDHB_EX10_R | 5’-CTGATGATTGCTGTGTCTTGG-3’ |  |  |
| 3’UTR | BCKDHB_EX11_F | 5’-AGCCAAGGTAGTGATGGTGG-3’ | 63ºC | 690 |
|  | BCKDHB_EX11_R | 5’-CATCCTGGTCATAAAGAACTGAAC-3’ |  |  |

AT: Annealing Temperature*;* F: Forward; R: Reverse*.*
