# Supplementary material for: Maple syrup urine disease in Brazilian patients: variants and clinical phenotype heterogeneity
Source: Orphanet J Rare Dis. 2020 Nov 1;15:309. doi: 10.1186/s13023-020-01590-7 (PMC7603684; doi:10.1186/s13023-020-01590-7)
Supplement: Supplementary file 3 — Additional file 3. Primers sequences, PCR conditions and amplified fragment sizes of the DBT gene. Description of data: Primer location, primer sequences, Annealing Temperature and amplified fragment sizes (amplicon size) of the DBT gene. [file 13023_2020_1590_MOESM3_ESM.docx]

Primers sequences, PCR conditions and amplified fragment sizes of the *DBT* gene.

| **Primer location** | **Primer Name** | **Primer sequence** | **AT** | **Amplicon size (bp)** |
| --- | --- | --- | --- | --- |
| Exon 1 | DBT_EX1_F | 5’-CTTCCCTCCCTATTGGTCG-3’ | 60ºC | 316 |
|  | DBT_EX1_R | 5’-CTCCGTTCTCTGCCCTTTATT-3’ |  |  |
| Exon 2 | DBT_EX2_F | 5’-CAGAAGGAATTTTGGGTAAGGA-3’ | 62ºC | 563 |
|  | DBT_EX2_R | 5’-CAATCTGGGCAACTGAGTGA-3’ |  |  |
| Exon 3 | DBT_EX3_F | 5’-GCCTCTGCCTGAGAACATTC-3’ | 60ºC | 376 |
|  | DBT_EX3_R | 5’-TGTATGACAAAGTCCTTCACCACT-3’ |  |  |
| Exon 4 | DBT_EX4_F | 5’-CTGAAAGTAAATGCTGGGCTAGA-3’ | 60ºC | 552 |
|  | DBT_EX4_R | 5’-ATCTCTTCCTTTTGCTATTGCCT-3’ |  |  |
| Exon 5 | DBT_EX5_F | 5’-CCCACTCTACCCATACCATTAGG-3’ | 60ºC | 606 |
|  | DBT_EX5_R | 5’-AGCACCTGACATAAGACCTGGT-3’ |  |  |
| Exon 6 | DBT_EX6_F | 5’-CCTGATGGTTACCACATGCA-3’ | 60ºC | 483 |
|  | DBT_EX6_R | 5’-TCTACTGAGGTAGCTTCCCCC-3’ |  |  |
| Exon 7 | DBT_EX7_F | 5’-GCAGTCAGTGTTCCAGCTTTG-3’ | 60ºC | 405 |
|  | DBT_EX7_R | 5’-CAAATAAATGTCCTACTCAAGCCTT-3’ |  |  |
| Exon 8 | DBT_EX8_F | 5’-GGAACTTTGGCTGGTCTGTATC-3’ | 60ºC | 660 |
|  | DBT_EX8_R | 5’-GCTGCTTCTTTTTGAGAGGGT-3’ |  |  |
| Exon 9-10 | DBT_EX9_10_F | 5’-ATGGCAGTGAAGGTTGATCC-3’ | 60ºC | 542 |
|  | DBT_EX9_10_R | 5’-TGTGTTTAGTCCCTGAATTTGCT-3’ |  |  |
| Exon 11 | DBT_EX11_F | 5’-GGTTTGCCTGATCTTACACCA-3’ | 60ºC | 469 |
|  | DBT_EX11_R | 5’-CCCAGGAGAACCATTACACC-3’ |  |  |

AT: Annealing Temperature*;* F: Forward; R: Reverse*.*
